# Modern PID/FOPID controllers for frequency regulation of interconnected power system by considering different cost functions

**DOI:** 10.1038/s41598-023-41024-5

**Published:** 2023-08-28

**Authors:** Mohamed Ahmed Ebrahim Mohamed, K. Jagatheesan, B. Anand

**Affiliations:** 1 Electrical Engineering Department, Faculty of Engineering at Shoubra, Benha University, Cairo, Egypt; 2 Department of Electrical and Electronics Engineering, Paavai Engineering College, Namakkal, Tamilnadu India; 3 Department of Electronics and Instrumentation Engineering, Hindusthan College of Engineering and Technology, Coimbatore, Tamilnadu India

**Keywords:** Engineering, Energy science and technology, Renewable energy

## Abstract

This article presents frequency regulation of an interconnected three-area power system (Thermal + Wind + Hydro). Fractional Order PID (FOPID) and Proportional-Integral-Derivative (PID) controllers are applied as subsidiary regulators to control the electrical power interconnected system at the time of sudden load variation. To accomplish this study, Genetic Algorithm (GA), Grey Wolf Optimizer (GWO), Sine Cosine Inspired Algorithm (SCIA) and Atom Search Inspired Algorithm (ASIA) are implemented to optimize the secondary regulators' gains (PID and FOPID) by considering various cost functions such as Integral Absolute Error (IAE), Integral Time Absolute Error (ITAE), Integral Square Error (ISE), and Integral Time Square Error (ITSE). Performance analysis in this work is conducted using various cost functions based on GA, GWO, SCIA and ASIA. The comparative analysis of the attained results reveals that GWO-PID and ASIA–PID settle at (83.83 s) and (30.31 s), respectively and ASIA-FOPID at (25.12 s). The controllers based on ITSE as a cost function outperform the comptrollers with other cost functions (ISE, IAE and ITAE). In addition, the ISE-based GA–PID and SCIA–PID settle at (113.92 s) and (35.1 s), respectively and SCIA-FOPID at (24.78 s). The ISE-based regulators yield improved response equated to other cost functions (ITSE, IAE and ITAE) optimized controllers. The robustness test also is carried out to validate the effectiveness of the proposed optimization techniques by changing the system parameters within ± 25% and ± 50% from their nominal values as well as changing the load pattern.

## Introduction

The primary aim of a power-generating system is to monitor and control the supply frequency, ensure stable power flow in the tie line of interconnected systems, maintain voltage profile and ensure stability of load flow conditions between areas within the desired value. The load-generation equilibrium ensures that customers are always provided with reliable and secure power. During nominal loading conditions, each power plant has self-control of stability and operating point. If sudden load demand arises, it will disturb the system supply frequency and power flow in the tie-line between interconnected power systems^1,2^. Thus, the frequency response contains oscillations that produce errors. To maintain the stability of the power generating system, a primary control loop (speed governor) and secondary controller are also introduced to ensure that the system parameters are kept within the specified limits^1,2^. The role of Automatic Generation Control (AGC) in a power system is to adjust the generated power output of several generators at dissimilar power generating plants, in response to modification in the load demand. In the AGC of the power system, area control error acts as the input for the controller to generate the required control signal that complies with the desired output response during the sudden load disturbance in the system.

Based on the literature review, it is found that many secondary controllers are introduced based on Bio- computation Inspired Algorithms (BIA). The authors of^3^ investigated the optimal gain value of PID controller using the Stochastic Particle Swarm Optimization technique for single-area AGC including Super Magnetic Energy Storage (SMES) and Redox Flow Battery (RFB) units with Integral Time Absolute Error (ITAE) and Integral Time Square Error (ITSE) cost functions. In addition, SPSO technique is proposed for tuning PID controller gain values for load frequency control of a single area reheat thermal power system^4^. Also, the authors of^5^ implemented SPSO algorithm for tuning the decentralized Load Frequency Control (LFC) of interconnected two-area thermal power structures. Moreover, a new variant of PSO had been deployed for controlling the frequency and voltage of multi-area power systems^6^. Harmony Search Algorithm (HSA) had been deployed for tuning PID controller of LFC problem^7^. Moreover, reduced order HSA is presented in^8^ to tune PID controllers for LFC of multi-area interconnected power systems. In addition, the authors of^9^ had been employed HSA based PID regulator for frequency control of multi-area systems including nonlinearities and boiler dynamics. Also, a Modified HSA (MHSA) is considered to tune PID regulator gain values for LFC of interconnected nonlinear thermal-hydro power networks^10^. In^11^, Bat Inspired Algorithm (BIA) is utilized to fine-tune the PI controller gain values for LFC of two area interconnected power networks. Hybrid Firefly and Pattern Search algorithm is executed to acquire optimal gain values of PI as well as PID regulators in^12^. Moreover, the performance of the proposed technique is compared with Bacteria Foraging Optimization Algorithm (BFOA), Genetic Algorithm (GA) and Ziegler Nichols (ZN) technique. Firefly Algorithm (FA) is executed to obtain PI/PID regulator gain values in multi-area LFC of grid-connected non-reheat electro-thermal power generating network and the performance response is equated with BFOA, GA, ZN, Differential Evolution (DE) and Particle Swarm Optimizer (PSO) based optimization methods in^13^. Neuro-Fuzzy Hybrid intelligent based PI regulator was considered for LFC of interconnected four-area power generating system in^14^. An emotional learning-based intelligent controller is proposed in^15^. Evolutionary computation techniques had been used for the control of single area power producing systems^16^. The design and modeling of various power system components with their behavior are given in^17^. LFC of 3-area organized power producing unit (Thermal, wind and hydro unit) is investigated with artificial intelligence technique tuned controller in^18^. In^19^, the author designed PSO based PI controller for LFC of three area interconnected systems to study AGC performance. The hydro-thermal interconnected nonlinear power system is examined by applying a fuzzy logic controller^20^ to improve the performance of the system. The LFC of the interconnected power system is investigated by considering the non-linearity effect of the Generation Rate Constraint (GRC) using the Hybrid Genetic-Firefly Algorithm (HGFA)^21^. Local Unimodal Sampling-TLBO (LUS-TLBO) optimization technique-based fuzzy PID controller is implemented in^22^. Self-adaptive modified bat algorithm has been implemented to acquire the proposed controller gain values of AGC controller for the grid-connected (four area) power network in^23^. Grey Wolf Optimization (GWO) algorithm-based classical controllers are introduced in^24^ multi-area interconnected (solar & thermal system) power generating units. The simulation behavior of the obtained results for GWO optimized controller is equated through GA, FA and PSO-tuned regulators. Many LFC-based optimization techniques are introduced such as classical^25^, fuzzy logic^26^, supervised artificial neural network^27^, GA^28^, PSO^29^, Artificial Bee Colony (ABC)^30^, FA^31^ and Cuckoo Search (CS)^32^ are available for the investigation of literature survey in LFC of interconnected power system. Using hybrid harmony search and cuckoo optimization, the Fuzzy PID controller is designed to resolve LFC problems in two area connected thermal (non-reheat) systems^33^. Moreover, Interval type 2 fuzzy PID with Big Bang Big-Crush^34^, fuzzy PID controller with filter^35^, Quasi-oppositional based learning (Q-OBL)^36^, Craziness based PSO (CPSO)^37^ are introduced as solutions for LFC challenges. In^38^, the author proposes Moth Swarm Algorithm (MSA) based PID controller for frequency stability of a hybrid power system with high wind power penetration in conjunction with Super Magnetic Energy Storage (SMES). In addition, the interior-point algorithm^39^, aggression method^40^, tracking approach^41^, fixed mode evaluation algorithm^42^, chaotic optimization algorithm^43^, and DE algorithm^44^ are executed to overcome LFC emergency in power systems. Dual gain scheduling PI regulator is demonstrated by applying a bat-inspired procedure for LFC of two area connected reheat thermal power network^11^. For LFC of interlinked two region interconnected power grid, the BFOA-developed PID controller is implemented considering nonlinearity effects^45^. FA constructed PI controller is designed for two area interconnected power system to solve LFC crisis which comprises Photovoltaic (PV) and thermal generation units^46^. AGC of a power system is investigated and rectified by applying multiple neural networks with an actor-critic strategy^47^. A multi-agent double deep Q network-action discovery (DDQN-AD) technique-based control strategy is applied for AGC of the power network in^48^. AGC crisis of interlinked power grid is analyzed by utilizing a deep reinforcement learning algorithm for overcoming crisis raised in the power grid^49^. Virtual generation ecosystem-based control strategy has been applied in the microgrid for solving AGC crisis and improving the superiority of produced power supply under sudden load demand conditions in the grid^50^. AGC issues of islanding smart networks have been overcome by implementing a wolf pack hunting strategy to solve AGC crisis of the grid understudy^51^. Multi-agent reinforcement learning-based scheme is applied for getting a smart control scheme in the power system network^52^. AGC issues in multi-area interlinked power systems are examined by applying a multi-step unified reinforcement learning scheme^53^. In^54^, the authors proposed a multiple-step greedy attribute and multiple-level allocation strategy for solving AGC issues in the power system.

The author proposes a novel hybridized harmony search algorithm designed fuzzy-3D controller in implementing LFC of a hybrid system that includes diverse energy sources in^55^, the power management of wind / solar integrated system is discussed with 3DOF-FOPID controller based on AI in^56^. Similarly, AGC of power generating unit is analyzed by designing and applying modified sine cosine algorithm-based fuzzy-aided PID controller^57^, deep Q-network-based type-II fuzzy controller^58^, hybridized GWO-SCA based type-II fuzzy controller^59^, improved-salp swarm optimized type-II fuzzy controller^60^ in LFC of AC microgrid system. By analyzing the above-published literature review, it is clearly shown that in recent days optimization techniques are mainly used for solving electric power system-related crises. Mainly applied for finding optimal values of parameters during emergencies. The main research gap in the literature is found that the previous work is not investigated with different controllers and various optimization techniques. Moreover, the authors of the presented article investigated the robustness of the suggested control techniques by changing load patterns and system parameters from their nominal values.

The key contributions provided by the authors of the proposed work are as follows:

1. Two modern optimization techniques namely, the Sine Cosine Inspired algorithm (SCIA) and Atom Search Inspired Algorithm (ASIA) are employed to design optimal LFC-based Proportional-Integral-Derivative (PID) and Fractional Order PID (FOPID) controllers with four different cost functions to obtain the most optimal controller gain values of the suggested interconnected power system.
2. Robustness test is carried out to validate the efficiency of the suggested optimization framework and the developed controllers by changing the load pattern and system parameter values from their nominal values.
3. A comprehensive comparative study of the applicable cost functions is synthesized to validate the efficiency of four different cost functions-based (GA-PID, GWO-PID, ASIA-PID and SCIA – PID, ASIA-FOPID and SCIA – FOPID) controllers response during sudden load demand in the proposed system.

The paper's organization is as follows: The introduction section gives crucial details about the present work and a literature review of various related works to resolve LFC/AGC crisis in multi-area connected power systems. The Proposed Simulink model that investigates the power generating system is given in the “Investigated power system model” section. The transfer function model and controller details and the objective function is given in “Design of controller and cost function”. Comparisons of the simulated response of PID and FOPID controllers tuned using different algorithms and cost functions are compared and discussed in “Simulation result and discussion” section and at the end; the “Conclusion” section summarizes the planned work’s performance in tuning controller gain values.

## Investigated power system model

**Figure 1:**
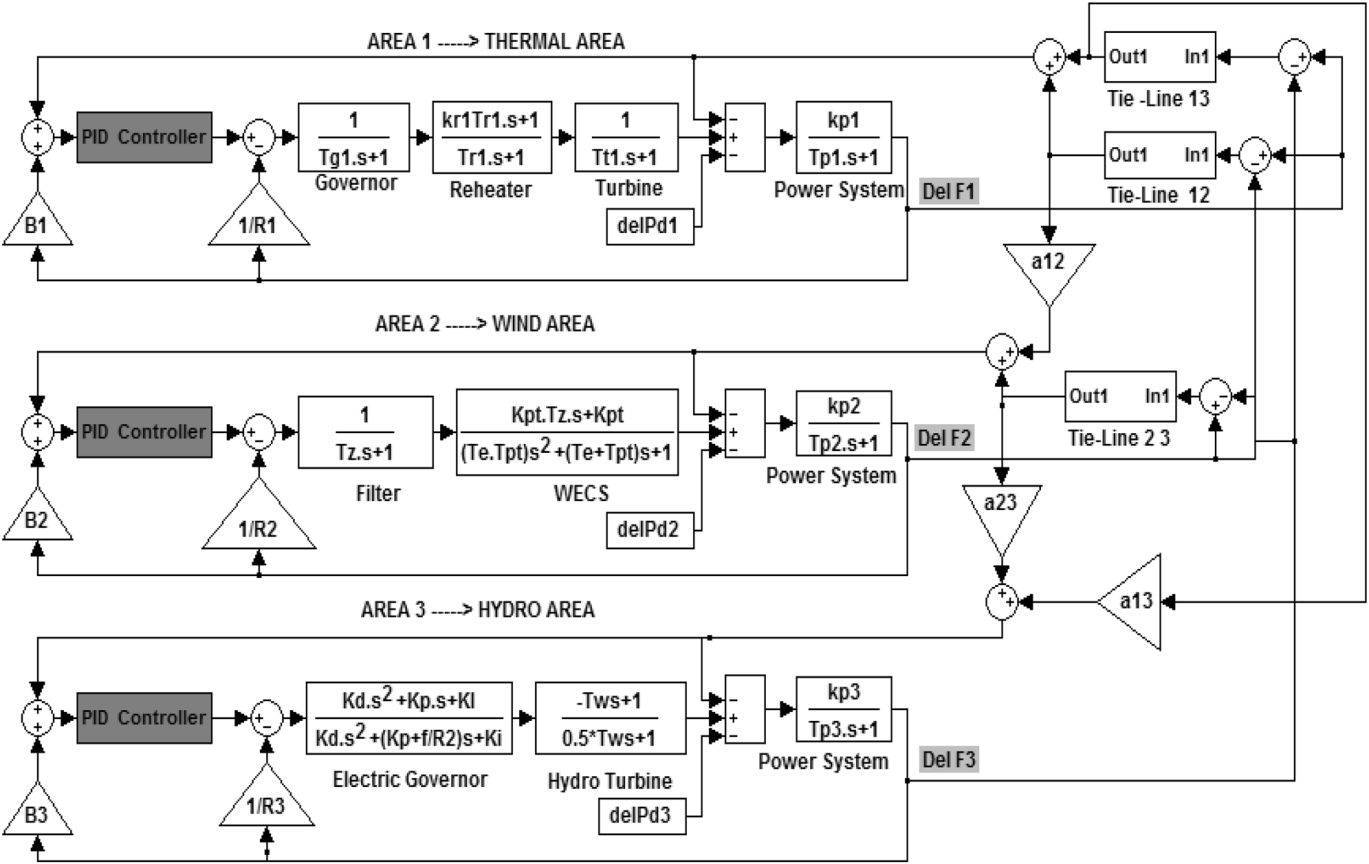
Three area interconnected power generating system Transfer function model with PID controller.

The proposed interconnected three-area power generating system is displayed in Fig. 1. It consists of thermal, hydro and wind power systems. That All three power-generating areas are organized with the support of a tie-line and are implemented as a subordinate controller to regulate power-generating system performance at the time of the unexpected load-changing scenario. Nominal parameter values of each proposed power-generating network are dispatched in^12–14^ and relevant abbreviations are given as follow:

f = 60 Hz, T_t_ = 0.3 s, T_g_ = 0.08 s, P_tie max_ = 200 MW, T_r_ = 10 s, K_p_ = 120 Hz/puMW, T_12_ = 0.544, H = 5 s, T_p_ = 20 s, P_r_ = 2000 MW, R = 2.4 Hz/p.u.MW, D = 0.00833 p.u.MW/Hz Density of air = 1.25 kg/m^3^, T_pt_ = 10.55 , Gear ratio = 70 , Radius of turbine blade = 45 m, Average wind velocity = 7 m/s, K_pt_ = 0.012, H = 5 s, T_i_ = 3 s, T_p_ = 20 s, K_r_ = 0.5.

The secondary PID controller input is Area Control Error (ACE) and it is defined as a linear grouping of errors in system frequency & errors in tie-line power flow changes. The output of the controller is u1, u2 and u3 control signals. The expression of the input signal is given in Eq. (1)–(3)^12,13^.

1 $${\mathrm{ACE}}_{1}={\mathrm{B}}_{1}{\Delta \mathrm{f}}_{1+}{\Delta \mathrm{P}}_{\mathrm{tie}12}$$

2 $${\mathrm{ACE}}_{2}={\mathrm{B}}_{2}{\Delta \mathrm{f}}_{2+}{\Delta \mathrm{P}}_{\mathrm{tie}13}$$

3 $${\mathrm{ACE}}_{3}={\mathrm{B}}_{3}{\Delta \mathrm{f}}_{3+}{\Delta \mathrm{P}}_{\mathrm{tie}23}$$

where ACE_1_, ACE_2_, ACE_3_ represent ACE in areas 1, 2 and 3. B_1_, B_2_ and B_3_ represent frequency bias constant in areas 1, 2 and 3 correspondingly. ∆f is denoted as frequency deviation in areas 1,2 and 3 respectively. ∆P_tie_ represents the deviation of power flow in the tie-line between connected power system areas 1 to 2, area 2 to 3 and area 3 to1.

## Design of controller and cost function

### PID controller

The PID controller output is the combination of proportional, integral, and derivative control actions^61^. The proportional controller takes care of and reduces the steady state error in system response^62–65^. The integral controller reduces the settling time and the derivative controller is responsible for the stability of the entire system at the time of emergencies^66–69^. This combination reduces the consequence of a disturbance and shortens the time it takes for the frequency level to return to its set point in all critical situations^70^. Moreover, PID is the most simple industrial controller for solving critical issues^6,71–75^.

**Figure 2:**
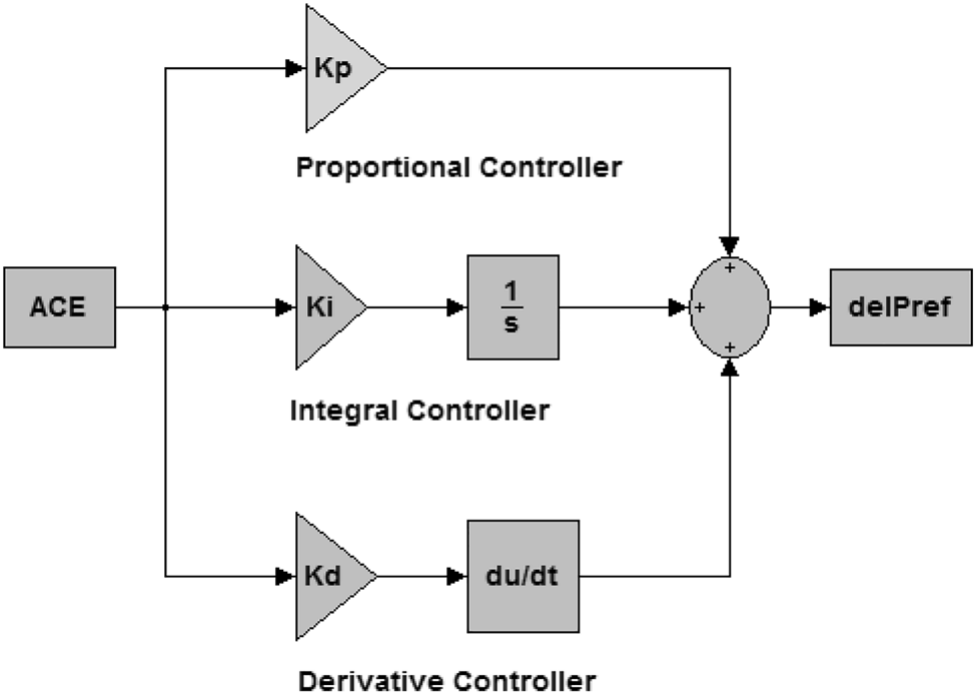
Arrangement of PID controller.

PID controller configuration is shown in Fig. 2. It contains three controllers namely, proportional, integral and derivative. The input of the secondary controller is area control error (ACE) and based on the error signal it generates the required control signal (delP_ref)_ to power generating system for frequency regulation. Therefore, the controller design with suitable gain values plays a vital part to achieve a healthier controller response during sudden load demand^13,15^.

### FOPID controller

The main modification between PID controller and FOPID controller is that the order of the FOPID controller is not an integer one^76–78^. Based on this characteristic, it provides an extra degree of freedom for tuning controller gain values and its performance is superior compared to conventional PID controller. Regarding the above characteristics of FOPID controller over conventional PID controller, it receives a considerable amount of attention over fast few years. Podlubny introduces the concept of FOPID controller in^76,77^. The transfer function of FOPID controller is given as follows:

4 $${G(s)}_{FOPID}= {K}_{p}+ \frac{{K}_{i}}{{S}^{\uplambda }}+{K}_{d}{S}^{\upmu }$$

**Figure 3:**
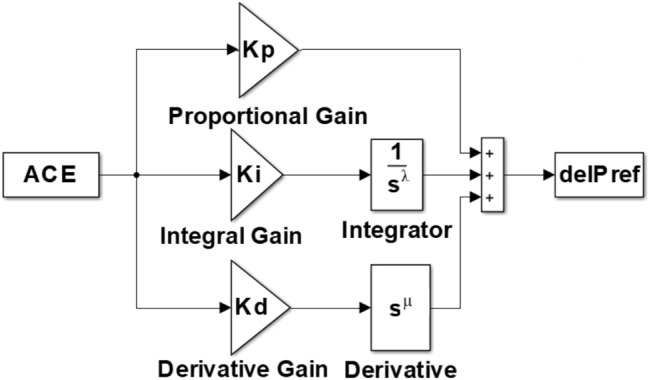
Arrangement of FOPID controller.

The gain values of proportional, integral and derivative controllers are represented as K_p_, K_i_ and K_d_. λ, µ represents the order of integral (I) and derivative (D) controller, respectively. Figure 3 demonstrates the structure of the proposed FOPID controller.

The dynamic response of the controller should provide quick relaxation time with minimal values of peak over and undershoot during sudden load demand. From the literature review, it is undoubtedly evident that many nature-inspired algorithms are presented to obtain optimal gain values of the controller. In this work, SCIA and ASIA optimization techniques are proposed to optimize controller gain values with four dissimilar cost functions and are compared with well-mature optimizers namely GA and GWO. The cost functions used are ISE, IAE, ITAE and ITSE. To design the regulator, the cost function is initially defined based on the essential description and constraint.

Expression for each cost function is given in Eq. (5)–(8)^13–15^.

Integral squared error

5 $$ J = \mathop \smallint \limits_{o}^{T} (\Delta f_{i,j} + \Delta P_{tie \rightleftarrows \rightleftarrows \rightleftarrows \;i,j} )^{2} dt $$

Integral time squared error

6 $$ J = \mathop \smallint \limits_{o}^{T} t.(\Delta f_{i,j} + \Delta P_{tie \rightleftarrows \rightleftarrows \rightleftarrows \;i,j} )^{2} dt $$

Integral absolute error

7 $$ J = \mathop \smallint \limits_{o}^{T} \left| {\Delta f_{i,j} + \Delta P_{tie \rightleftarrows \rightleftarrows \rightleftarrows \;i,j} } \right|dt $$

Integral time absolute error

8 $$ J = \mathop \smallint \limits_{o}^{T} t.\left| {\Delta f_{i,j} + \Delta P_{tie \rightleftarrows \rightleftarrows \rightleftarrows \;i,j} } \right|dt $$

where J = performance index, t = simulation time, ∆f = frequency deviations, ∆P_tie_ = tie-line power deviations.

## Meta-heuristic optimization techniques (MOTs)

In AGC of the power system, area control error acts as the input for the controller to generate the required control signal that complies with the desired output response during the sudden load disturbance in the system. The simulation process ended after reaching maximum iteration and the system yield a better response at the time of sudden load demand situations in the power system. Different MOTs have been developed and applied in recent years to address numerous dynamic computational issues and provide solutions for LFC problems^79^. Moreover, MOTs have better flexibility compared to conventional optimization methods and it deals with various complex optimization problems effectively such as blade pitch control problem in wind energy field^71,80–84^, maximum power point tracking in photovoltaic-based energy field^85–95^ and energy management schemes in renewable energy-based electrical power systems^96–100^.

Despite MOTs being utilized in various applications. However, there is no single MOT that can solve all optimization problems^24,25^. A population-based meta-heuristic approach, which is the actual GWO value, is suggested in^101^. It can be successfully extended to many functional implementations due to the GWO algorithm's simplicity, consistency, and efficiency^101^. Many improvements have subsequently been made to the original GWO algorithm. These modifications, however, use binary or decimal encoders to place the GW; information on the individual genes is also minimal. The simple GWO, therefore has recently been suggested as a modern MOT to solve numerous optimization problems^102,103^.

In the proposed research work, GWO technique is developed to obtain optimal controller gain values in LFC of a multi-area power-generating system network. GWO technique is used to solve nonconvex engineering optimization problems. In nature, it replicates the social stratum and chasing system of grey wolves. The grey wolves are split into four primary levels. Leaders are the first-level wolves and their duty is decision-making. Decisions or other actions are made by the second-tier wolves. Scouts, sentinels, elders, hunters, and caretakers are the third-level wolves who perform orders but can direct other underlying entities and their duties. In all the wolves, in the fourth stage wolves act as an executor.

Optimized gain values are collected by applying four types of cost functions which are IAE, ITAE, ISE and ITSE. The performance of GWO-PID controller is verified by comparing it against GA-PID controller’s performance based on the same system^18^. The details of the proposed algorithm and optimized gain values are given in the following section.

### Grey Wolf optimization

The Grey Wolf Optimization technique is a most recently developed MOTs based on the hunting behavior and leadership hierarchy nature of grey wolves. GWO is the freshest metaheuristics swarm intelligence computational method. Due to its attractive characteristics over other swarm computational intelligence techniques. In addition, it is simple to use, more flexible and scalable. Due to these reasons, GWO has nowadays gained an enormous research interest with tremendous listeners from numerous domains in engineering. There are a greater number of factors considered at the time of developing a genetic algorithm for optimization. Many normal parameters value can be customized to affect the performance. Such as variable specification, tight variable bounds, weighting strategies and constraints. which may slow down the optimization process.

The major role of MOT algorithms is to find global optima while avoiding being stuck in local optima. The proposed GWO technique is robust, simple and it has been developed to solve various complex optimization issues^101–103^. In GWO technique, the group of grey wolves is classified into four different groups such as alpha (α), beta (β), omega (ω) and delta (δ).

The hunting manners are divided into the following three steps:

Step 1: Tracking, Hurtling and approaching the prey.

Step 2: Surrounding and distressing prey till it stops moving.

Step 3: Aggressive prey.

The surrounding behavior is represented by the following mathematical expression^101^:

9 $$\overrightarrow{D}=\left|\overrightarrow{C}.\overrightarrow{{X}_{P}}\left(t\right)-\overrightarrow{X}(t)\right|$$

10 $$\overrightarrow{\mathrm{X}}\left(\mathrm{t}+1\right)= {\overrightarrow{\mathrm{X}}}_{\mathrm{p}}\left(\mathrm{t}\right)-\overrightarrow{\mathrm{A}}.\overrightarrow{\mathrm{D}}$$

In these above equations $\overrightarrow{{X}_{P}}$—indicates the prey position vector,—$\overrightarrow{\mathrm{X}}$signifies the grey wolf position vector, $\overrightarrow{\mathrm{A}}$ and $\overrightarrow{\mathrm{C}}$ vectors are coefficients and t represents the current iteration.

11 $$\overrightarrow{\mathrm{A}}=2\overrightarrow{a}.{\overrightarrow{\mathrm{r}}}_{1}-\overrightarrow{\mathrm{a}}$$

12 $$\overrightarrow{\mathrm{C}}=2.{\overrightarrow{\mathrm{r}}}_{2}$$

$\overrightarrow{\alpha }$ Value decreases linearly from 2 to 0 during iteration and r_1_, and r_2_ indicate the random numbers which lie in the range of 0 to 1. During the optimization process, values of $\omega$ wolves revise their positions around $\alpha$, $\beta$ and $\delta$. Based on these values,$\omega$ wolves are repositioned.

The updated mathematical models of wolves’ positions are determined as per the following^20–22^:

13 $${\overrightarrow{D}}_{\alpha }=\left|{\overrightarrow{C}}_{1}.{\overrightarrow{X}}_{\alpha }-\overrightarrow{X}\right|$$

14 $${\overrightarrow{D}}_{\beta }=\left|{\overrightarrow{C}}_{2}.{\overrightarrow{X}}_{\beta }-\overrightarrow{X}\right|$$

15 $${\overrightarrow{D}}_{\delta }=\left|{\overrightarrow{C}}_{3}.{\overrightarrow{X}}_{\delta }-\overrightarrow{X}\right|$$

16 $${\overrightarrow{X}}_{1}={\overrightarrow{X}}_{\alpha }-{\overrightarrow{A}}_{1}. {\overrightarrow{D}}_{\alpha }$$

17 $${\overrightarrow{X}}_{2}={\overrightarrow{X}}_{\beta }-{\overrightarrow{A}}_{2}. {\overrightarrow{D}}_{\beta }$$

18 $${\overrightarrow{X}}_{3}={\overrightarrow{X}}_{\delta }-{\overrightarrow{A}}_{3}. {\overrightarrow{D}}_{\delta }$$

19 $$\overrightarrow{\mathrm{X}}\left(\mathrm{t}+1\right)= \frac{{\overrightarrow{X}}_{1}+{\overrightarrow{\mathrm{X}}}_{2}+ {\overrightarrow{\mathrm{X}}}_{3}}{3}$$

where $\overrightarrow{{X}_{\alpha }}$, $\overrightarrow{{X}_{\beta }}$, $\overrightarrow{{X}_{\delta }}$ are signifies the position of the, β, and δ respectively, $\overrightarrow{{C}_{1}}$, $\overrightarrow{{C}_{2}}$, $\overrightarrow{{C}_{3}}$ and $\overrightarrow{{A}_{1}}$, $\overrightarrow{{A}_{2}}$, $\overrightarrow{{A}_{3}}$ represent vectors,$\overrightarrow{(X})$ indicates the current solution position and the number of iterations is represented by (t). Then, based on the position’s values of α, β and δ the ω wolves update their positions regularly.

The $\overrightarrow{A}$ which represents the random vector and $\overrightarrow{C}$ which represents the adaptive vector used to assist the algorithm with the local optimization value (prey). The half of iterations are committed to investigation, if |A > 1|, the value of A is decreased from A > 1 over the course of the iteration. The value of C is in the iteration value range of 2 to 0. When the value of C is greater than 1, the vector C starts exploration. In between, the remaining other half of the iterations are dedicated to exploitation when |A|< 1 exists. When the condition C < 1 occurs, exploitation is confirmed^101,102^.

The steps followed by GWO algorithm during PID controller gain value optimization are as follows^102–104^.

Step 1: Start the process.

Step 2: Initialize GWO population.

Step 3: Calculate the fitness function of each search agent.

Step 4: Update the position of the current agent a, A and C.

Step 5: All search agents’ fitness is calculated.

Step 6: Check if the satisfied condition is reached or not.

Step 7: Go to Step 4 if, No and repeat.

Step 8: Display optimal PID gain values, if Yes.

Step 9: Stop.

### Sine cosine inspired algorithm

Mirjalili^104^ developed a sine cosine-inspired algorithm based on the sine cosine laws. The random solutions are generated automatically in the first population (controller gain values in the form of a variable vector) by the software. The best locations are defined based on the fitness function with the help of search agents for the minimization of area control error. With the support of this new position P is obtained by assessing the entire population in search agent.

The mathematical modeling of SCIA technique is developed based on search agent updating^104^, as depicted in the below Eq. (20).

20 $${X}_{i}^{t+1}=\left\{\begin{array}{c}{X}_{i}^{t}-({R}_{1}\mathrm{sin}\left(2\pi {R}_{2}\right) \left|{2R}_{3}{P}_{i}^{t}-{X}_{i}^{t}\right| k\le 0.5\\ \\ {X}_{i}^{t}-({R}_{1}\mathrm{cos}\left(2\pi {R}_{2}\right)\left|{2R}_{3}{P}_{i}^{t}-{X}_{i}^{t}\right| k>0.5\end{array}\right.$$

In the above equation k,${R}_{2}$ & ${R}_{3}$ are randomly generated random values, in addition ${R}_{1}$ is calculated with the support of Eq. (21).

21 $${R}_{1}=S-t\frac{S}{{t}_{max}}$$

In the above $t$ and ${t}_{max}$ is pointing out the iterations number and maximum value, whereas $S$ represents constant value.

**Figure 4:**
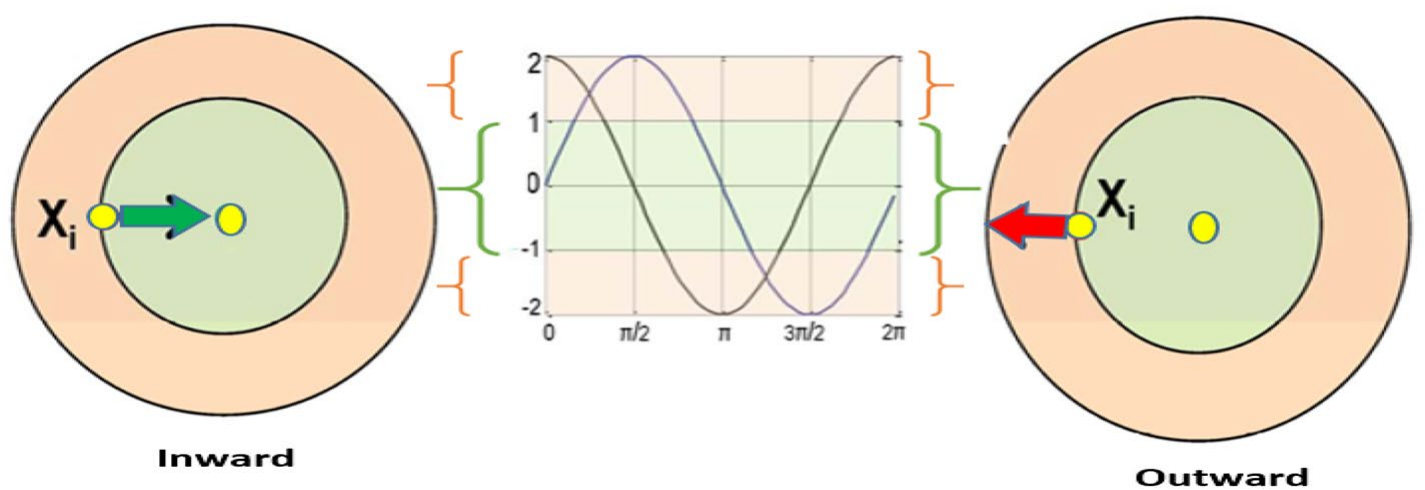
SCIA Searching sub-areas.

Figure 4 denotes SCIA technique and its methodology that depends on a circular pattern. In that center of the circle, it is denoted that the best solution and remaining feasible solutions are available outside. The limitations and constraints for operations are represented in the border of the circle. The variable vector upper and lower limits are denoted in the borders of each segment. In addition, two sub-areas are divided and assumed to discover potential areas of Xi solutions.

The motion direction of X_i^ ( P position is outward when R_1 value < 1 and inward when R_1 is > 1) is defined using control factor R1. Based on the R1 and R2 values the inward and outward movements of Xi are determined within the range of 0 to" 2π". Within those probabilistic weights, P is defined using R3. Also switching factor k between two parts (sine and cosine) is determined randomly as per Eq. (20) and the random number Ki is (0:1).

**Figure 5:**
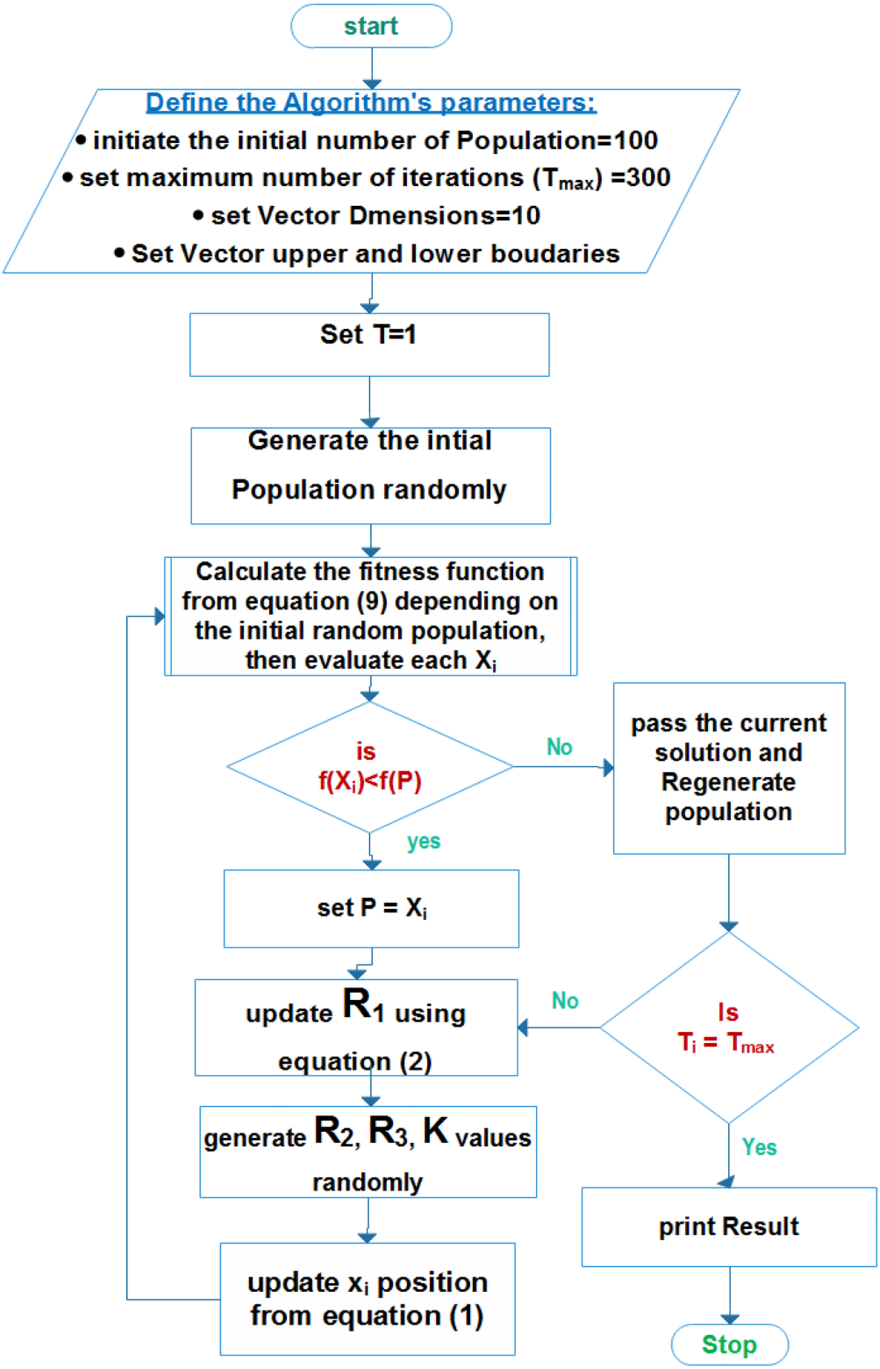
SCIA Optimization Algorithm.

At the time of Xi movement, the boundaries are defined in the range of [-1, + 1] to achieve Pbest position and each search space exploited by the algorithm. The search space is exploited using Pbest position. The steps involved in SCIA methodology are clearly shown in the below flow chart Fig. 5.

### Atom search inspired algorithm

Based on the behavior of atomic dynamics, the atom search-inspired algorithm (ASIA) is developed in 2018 by Zhao et al.^105^. ASIA is developed to mimic the atomic motion model because it follows certain molecular mechanics standards. In the atmosphere, all substances include atoms. Atoms are bounded by bonds (covalent) and transformed into molecules. There are two types of forces generated within the atom, such as attractive and repulsive forces. The interactions and forces are developed because of the gap between atoms. Whenever the gap between atoms decreases equally repulsive force increases between them. The frequency of atoms increases when the gap between atoms increases^105–108^. By using the constraint force (CF), the flow of motion of the atom is controlled and with the support of interactive forces (IF), the motion action is induced in atoms. The value of acceleration is calculated by applying the second law of Newton and it is given by:

22 $${\alpha }_{i}= ({F}_{i}+{G}_{i})/{m}_{i}$$

**Figure 6:**
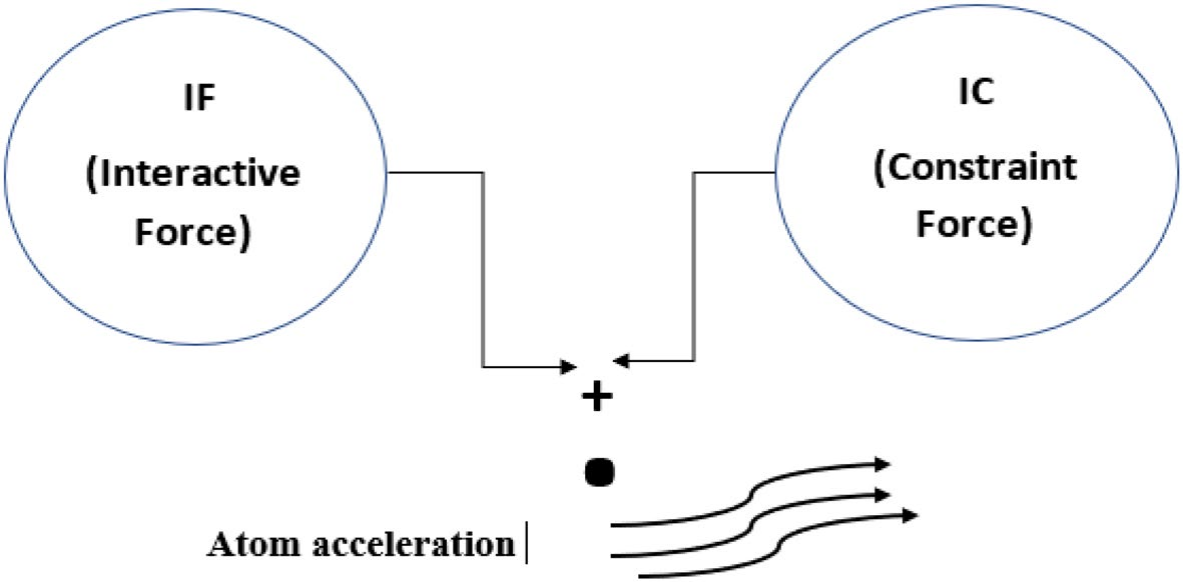
The motion of the atom due to CF and IF.

In the above equation ${\alpha }_{i}$ signifies the acceleration of ith atom, ${F}_{i}$ printouts the interaction force, ${G}_{i}$ indicates constraint force and ${m}_{i}$ represents the mass of the ith atom. The motion of the atom due to forces is shown in Fig. 6^108^.

The velocity and position of ith atom at (t + 1) iteration are given by^105–108^:

23 $${{Velocity}_{i}}^{d(t+1)}= {rand}^{d} {{Velocity}_{i}}^{d(t+1)}+ {{A}_{i}}^{d(t)}$$

24 $${{atom}_{i}}^{d(t+1)}= {{atom}_{i}}^{d(t)}+ {{velocity}_{i}}^{d(t+1)}$$

The enhancement of exploration and exploitation at the time of the initial and final process of iterations had been validated by the authors of^105–108^.

The tuning of the controller gain values by applying ASIA techniques follows the following phases:

Phase 1: Start.

Phase 2: Initialize atoms' position and velocity.

Phase 3: Check criterion is reached or not. If yes go to phase 9. Otherwise, go to the next phase.

Phase 4: Calculate atoms fitness and best atom.

Phase 5: Calculate the mass of the atom.

Phase 6: Find K neighbors for each atom.

Phase 7: Calculate the force (interaction and constraint).

Phase 8: Update the position of the atom.

Phase 9: Update the velocity of the atom.

Phase 10: Return to the best atom.

Phase 11: Stop.

**Figure 7:**
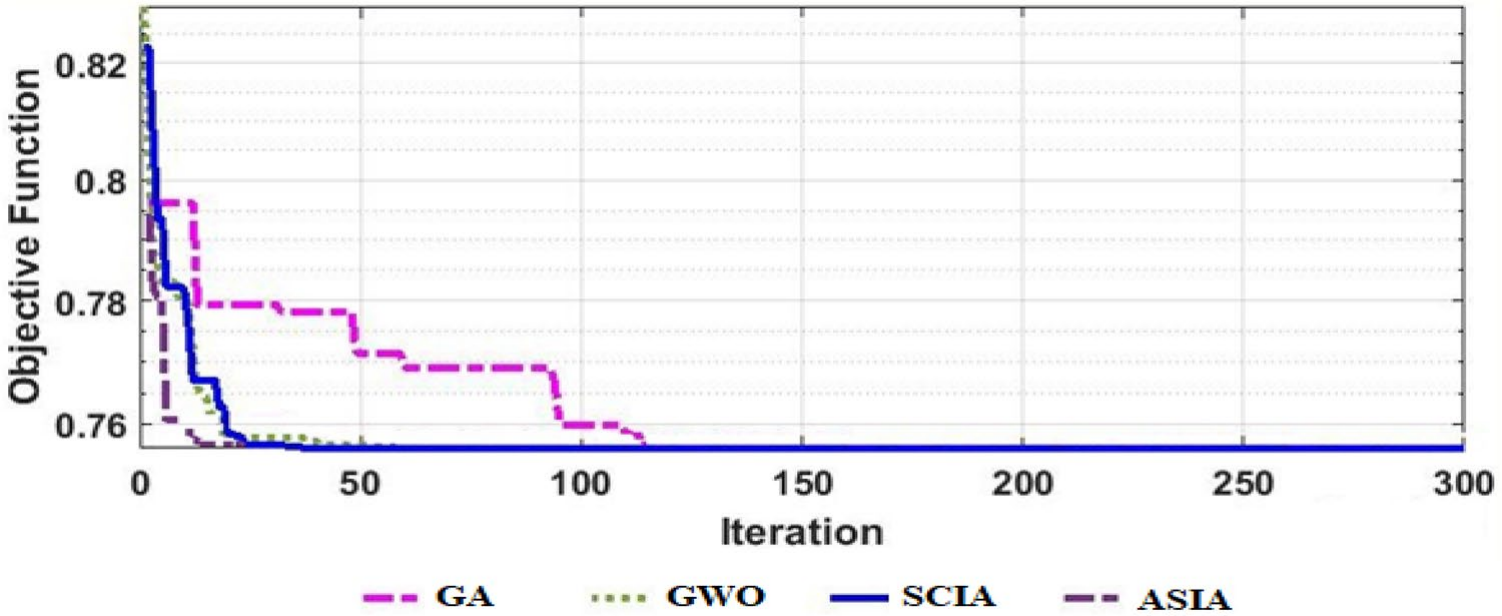
convergence curves of the applied optimization techniques for ITSE cost function.

Figure 7 shows the convergence curves for applying the optimization methodologies ASIA, SCIA, and GWO in addition to GA with the pre-specified constraints on the interconnected power system using ITSE cost function. From this figure, it is noted that for the same number of iterations (300) and the same number of search agents (100), ASIA converges faster than SCIA, GWO and GA methodologies in terms of minimum iterations number and low computation time. Moreover, ASIA needs only 33 iterations, whereas SCIA, GWO and GA require 41, 57 and 137 iterations to reach the optimal objective function value, respectively.

By applying ASIA technique, the controller gain values are optimized and the respective gain values are listed in Tables 4 and 6. The GA-PID, GWO-PID, ASIA-PID and SCIA-PID controllers with various objective functions are dispatched in Tables 1, 2, 3 and 4. The gain parameters of ASIA-FOPID and SCIA-FOPID controllers are given in Tables 5 and 6.

**Table 1:** Parameters of PID controller by applying GA method with various cost functions.

Cost function/controller gain	GA			
	IAE	ISE	ITAE	ITSE
Kp1	− 1.2077	− 0.3623	− 1.2639	− 0.6528
Ki1	− 0.1296	− 0.4369	− 0.2782	− 0.3828
Kd1	− 1.4253	− 1.0438	− 1.5719	− 1.6603
Kp2	− 0.8588	− 0.9991	− 1.6123	− 1.98
Ki2	− 0.3143	− 0.4292	− 0.3633	− 0.2841
Kd2	− 0.9982	− 1.0593	− 0.5691	− 0.0228
Kp3	− 0.5880	− 1.3855	− 1.0372	− 0.9267
Ki3	− 0.1848	− 0.0082	− 0.2240	− 0.1681
Kd3	− 0.8567	− 0.4770	− 0.5023	− 1.2302
Settling time (s)	153.1791	**113.9218**	130.1834	132.6339

Significant values are in [bold].

**Table 2:** Parameters of PID controller by applying GWO method with various cost functions.

Cost function/controller gain	GWO			
	IAE	ISE	ITAE	ITSE
Kp1	− 0.5074	− 1.6995	− 0.8044	− 0.8177
Ki1	− 0.0104	− 0.2139	− 0.1068	− 0.3081
Kd1	− 1.3125	− 1.7371	− 1.9677	− 0.2504
Kp2	− 1.1145	− 1.5442	− 1.9083	− 0.9705
Ki2	− 0.2475	− 0.2365	− 0.2388	− 0.1893
Kd2	− 1.7634	− 0.0026	− 1.5031	− 0.7088
Kp3	− 0.0551	− 1.5897	− 0.8096	− 1.2603
Ki3	− 0.0835	− 0.0775	− 0.2898	− 0.0676
Kd3	− 1.5751	− 0.6098	− 1.3102	− 0.9499
Settling time (s)	155.2114	133.7831	295.4874	**83.7516**

Significant values are in [bold].

**Table 3:** Parameters of PID controller by applying SCIA method with various cost functions.

Cost function/controller gain	SCIA			
	IAE	ISE	ITAE	ITSE
Kp1	1.97	1.58	1.80	1.48
Ki1	0.42	0.19	0.48	0.45
Kd1	1.33	0.52	0.87	0.96
Kp2	1.29	1.28	0.37	0.95
Ki2	0.23	0.40	0.27	0.38
Kd2	1.88	1.48	1.55	1.43
Kp3	1.49	1.21	0.44	0.09
Ki3	0.08	0.08	0.03	0.03
Kd3	1.68	0.58	0.48	1.26
Settling time (s)	45.6	**35.1**	42.6	48.4

Significant values are in [bold].

**Table 4:** Parameters of PID controller by applying ASIA method with various cost functions.

Cost function/controller gain	ASIA			
	IAE	ISE	ITAE	ITSE
Kp1	0.95	1.40	1.70	1.34
Ki1	0.22	0.18	0.35	0.23
Kd1	1.02	0.48	0.55	1.24
Kp2	1.96	0.70	0.13	1.94
Ki2	0.34	0.45	0.17	0.06
Kd2	0.42	0.80	0.82	0.36
Kp3	0.51	0.16	0.25	0.40
Ki3	0.04	0.01	0.10	0.02
Kd3	1.59	0.28	0.89	1.72
Settling time (s)	65.1	64.1	42.8	**30.31**

Significant values are in [bold].

**Table 5:** Optimized gain values of FOPID controller by utilizing SCIA method with various cost functions.

Cost function/controller gain	SCIA			
	IAE	ISE	ITAE	ITSE
Kp1	0.25	1.85	0.64	0.31
Ki1	0.21	0.48	0.47	0.10
Kd1	0.36	0.46	1.81	0.60
λ1	0.90	0.78	0.77	0.95
μ1	0.68	0.92	0.86	0.85
Kp2	0.19	0.16	0.58	0.53
Ki2	0.17	0.04	0.49	0.04
Kd2	0.21	0.29	1.23	0.05
Λ2	0.75	0.45	0.82	0.63
μ2	0.23	0.63	0.92	0.63
Kp3	0.87	0.13	0.19	0.72
Ki3	0.06	0.18	0.49	0.33
Kd3	1.79	1.28	1.96	0.16
λ3	0.40	0.54	0.41	0.36
μ3	0.42	0.77	0.70	0.30
Settling time (s)	25.69	**24.78**	25.30	25.05

Significant values are in [bold].

**Table 6:** Optimized gain values of FOPID controller by utilizing ASIA method with various cost functions.

Cost function/controller gain	ASIA			
	IAE	ISE	ITAE	ITSE
Kp1	1.28	0.52	1.39	1.34
Ki1	0.09	0.39	0.25	0.26
Kd1	1.98	0.15	0.62	0.74
λ1	0.94	0.8	0.94	0.83
μ1	1	0.84	0.63	0.73
Kp2	1.61	1.72	1.43	0.64
Ki2	0.04	0.31	0.32	0.38
Kd2	1.34	1.95	1.14	0.23
Λ2	0.95	0.1	0.95	0.99
μ2	0.39	0.48	0.11	0.82
Kp3	0.17	1.3	0.8	1.49
Ki3	0.3	0.38	0.13	0.11
Kd3	0.07	1.05	0.83	1.27
λ3	0.2	0.45	0.36	0.03
μ3	0.39	0.13	0.54	0.57
Settling time (s)	28.1	26.98	27.59	**25.12**

Significant values are in [bold].

## Simulation result and discussion

The model (Simulink) of the proposed power-generating network is designed and demonstrated by using MATLAB/SIMULINK environment by considering PID and FOPID controllers as secondary controllers. The PID controller’s gain values are optimized using SCIA, ASIA, GWO and GA algorithms with four different cost functions by considering 1% sudden load pattern (SLP) in area 1 is given in Tables 1, 2, 3, and 4. Similarly, FOPID controller parameters (gain values) are optimized by applying SCIA, ASIA technique with four dissimilar cost functions. The simulation results are compared and evaluated in the following sections:

### Analysis of GA-based PID controller behavior

**Figure 8:**
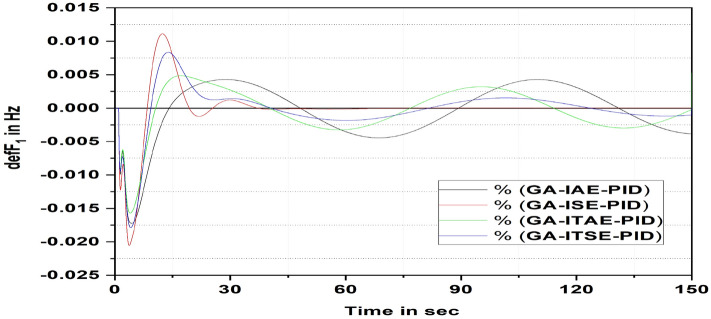
Area 1 Frequency deviation comparisons of GA PID controller response with dissimilar cost functions.

Figure 8 illustrates the response of area 1 (thermal) frequency deviations by considering GA-optimized gain values with four different cost functions. Based on the comparison, it is undeniably observed that compared to other cost features, the PID controller with ISE provides a quicker settled response.

Further investigation shows that ISE cost function with GA-tuned controller response settles faster compared to other cost functions. The system responses are yields minimum damping oscillation during sudden load demand.

**Figure 9:**
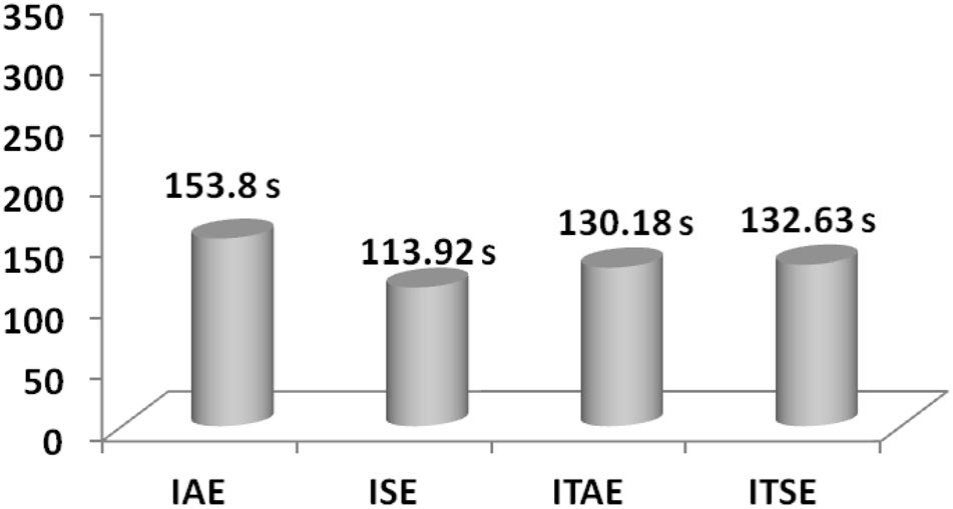
Settling time comparisons for GA PID controller with dissimilar cost functions.

Figure 9 shows a relaxation time comparison. It is noted that the GA-PID controller response based on ISE settles faster compared to the GA-PID controller response based on other cost functions.

Table 7 represents the percentage of improvement of ISE-based GA-PID controller over IAE, ITAE and ITSE-based GA-PID controller at the time unexpected load demand conditions.

**Table 7:** Percentage of improvement of ISE-based GA-PID controller over other cost functions.

Cost function	Relaxation time (s)	Percentage of improvement (%)
IAE	153.8	25.93
ITAE	130.18	12.49
ITSE	132.63	14.11

Based on the values tabulated in Table 7, it is evident that ISE constructed GA-PID controller improves system performance during emergency load demand conditions.

### Analysis of GWO-tuned PID controller behavior

**Figure 10:**
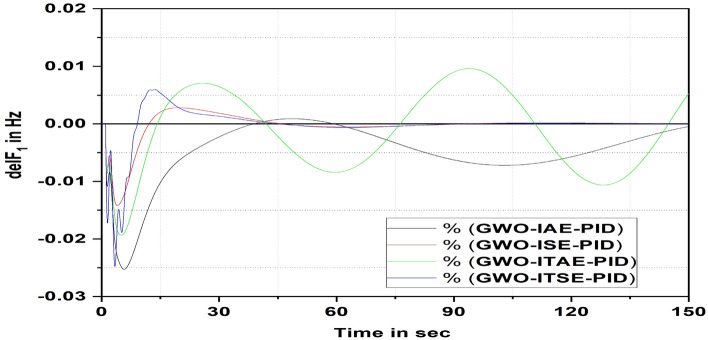
Area 1 Frequency deviation comparisons of GWO-tuned PID controller response with different cost functions.

Figure 10 represents the deviations in frequency by considering GWO algorithm-tuned PID controller with four cost functions. Based on the results, it is detected that ISE—GWO-PID controller provides a fast response and settles with lesser peak overshoot and undershoot value compared to ITSE, IAE, and ITAE-based PID controllers.

**Figure 11:**
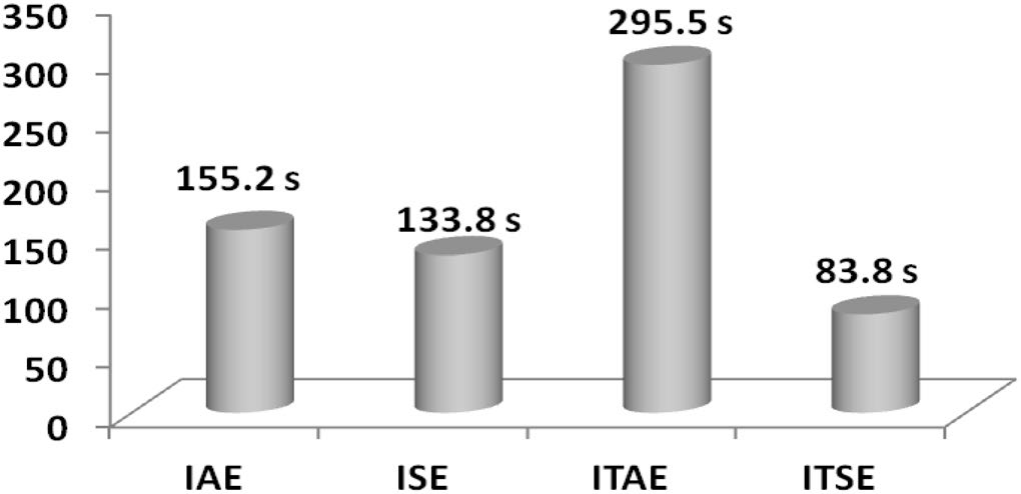
Settling time comparisons for GWO-PID (IAE, ISE, ITAE & ITSE) controller.

Figure 11 displays the relaxation time comparison plot and it is apparent that the ITSE-based GWO-PID controller response settles faster than the ISE, IAE, and ITAE-PID-based controllers’ response.

Table 8 provides a performance analysis of ITSE cost function tuned GWO-PID controller over the other three cost functions (ISE. IAE, ITAE) based GWO-PID controller response during unexpected load variation in the grid-connected power system. The values tabulated in Table 8 justify that ITSE-based GWO-PID controller gives superior results over ISE, IAE, ITAE based controllers.

**Table 8:** Enhancement of ITSE-based GWO-PID controller over other cost functions.

Cost function	Relaxation time (s)	Percentage of improvement (%)
IAE	155.2	46.01
ISE	133.8	37.37
ITAE	295.5	71.64

### Analysis of SCIA-tuned PID and FOPID controller behavior

The behavior of SCIA technique tuned PID and FOPID controller is analyzed in this section under 1% SLP in area 1 with four different cost functions.

**Figure 12:**
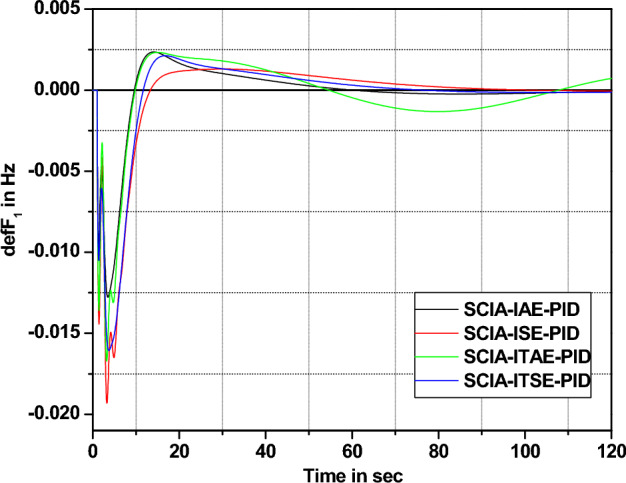
Area 1 Frequency deviation comparisons of SCIA-tuned PID controller response with different cost functions.

**Figure 13:**
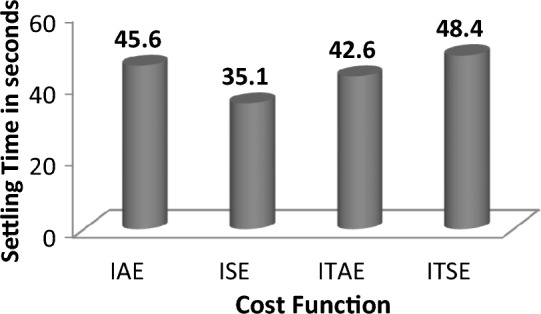
Settling time comparisons for SCIA-tuned PID controller with different cost functions.

Analyzing the response comparisons in Fig. 12 and bar chart comparisons in Fig. 13 effectively depicted that ISE cost function utilized SCIA technique tuned controller provides better controller performance compared to additional cost functions (IAE, ITSE and ITAE). Moreover, Table 9 validate the Percentage of improvement of ISE-based SCIA-PID controller over other cost functions.

**Table 9:** Percentage of improvement of ISE-based SCIA-PID controller over other cost functions.

Cost function	Relaxation time (s)	Percentage of improvement (%)
IAE	45.6	23.03
ITAE	42.6	17.61
ITSE	48.4	27.48

**Figure 14:**
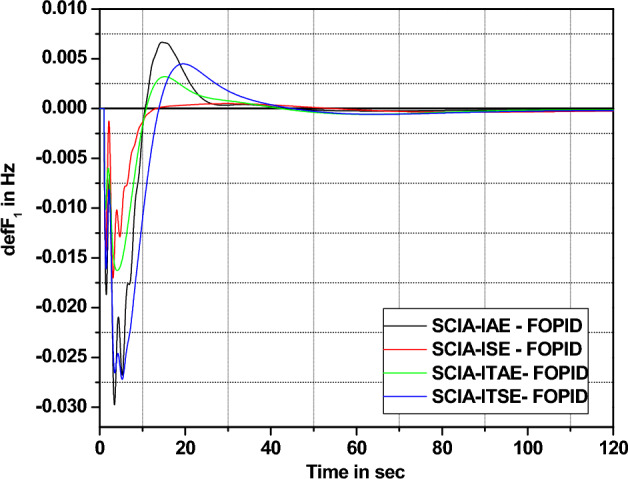
Area 1 Frequency deviation comparisons of SCIA-tuned FOPID controller response with different cost functions.

**Figure 15:**
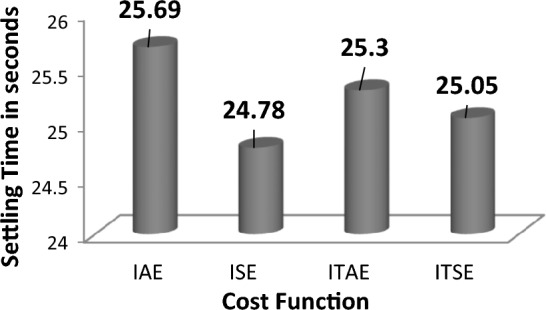
Settling time comparisons for SCIA-tuned FOPID controller with different cost functions.

The different functions created FOPID controller behavior in Fig. 14 and numerical values comparisons in bar chart Fig. 15 proved that ISE cost functions based FOPID controller get a more superior response over the other cost functions utilized controller response. Also, the improvement of ISE cost function-based response in terms of settling time is given in Table 10.

**Table 10:** Percentage of improvement of ISE-based SCIA-FOPID controller over other cost functions.

Cost function	Relaxation time (s)	Percentage of improvement (%)
IAE	25.69	3.54
ITAE	25.3	2.06
ITSE	25.05	1.08

### Analysis of ASIA-tuned PID and FOPID controller behavior

ASIA optimization technique is applied to tune the gain values of PID and FOPID controllers in frequency regulation of interconnected power systems with four different cost functions. The response comparisons of ASIA technique-tuned PID and FOPID controllers are investigated in this section.

**Figure 16:**
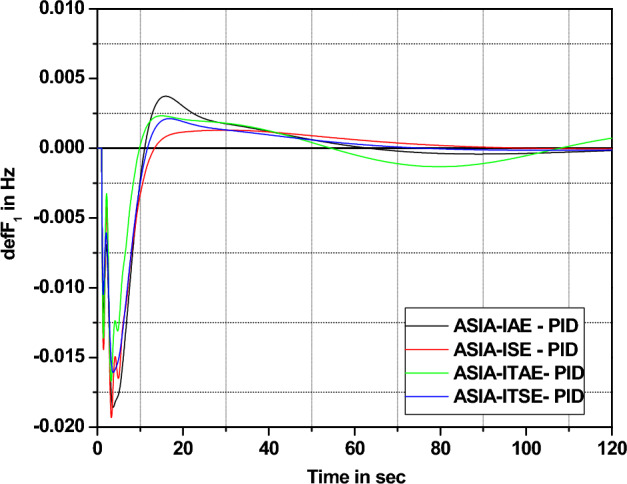
Area 1 Frequency deviation comparisons of ASIA-tuned PID controller response with different cost functions.

**Figure 17:**
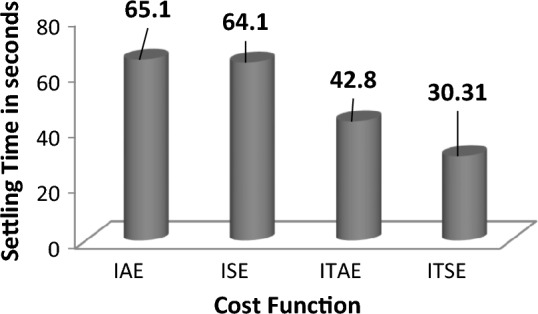
Settling time comparisons for ASIA-tuned PID controller with different cost functions.

The response assessments in Fig. 16 effectively show that ITSE-based ASIA PID controller provides more enhanced response remaining functions (cost) employing minimal settling time. Also, respective numerical values of improvement for ITSE-based ASIA-FOPID controller over other functions are highlighted in Fig. 17 and tabulated in Table 11.

**Table 11:** Percentage of improvement of ISE-based GA-PID controller over other cost functions.

Cost function	Relaxation time (s)	Percentage of improvement (%)
IAE	65.1	53.44
ISE	64.1	52.71
ITAE	42.8	29.18

**Figure 18:**
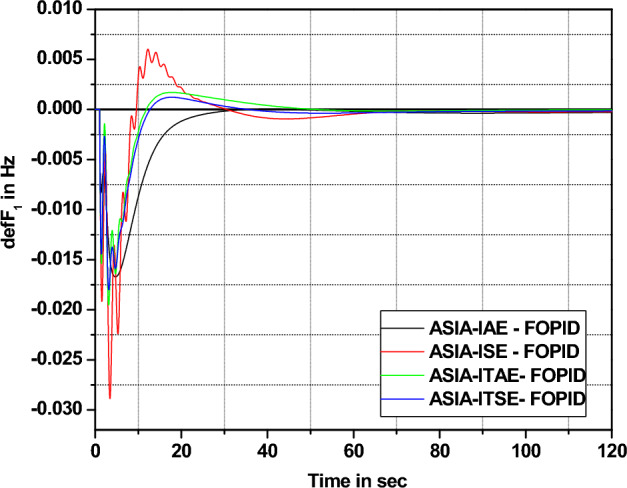
Area 1 Frequency deviation comparisons of ASIA-tuned FOPID controller response with different cost functions.

**Figure 19:**
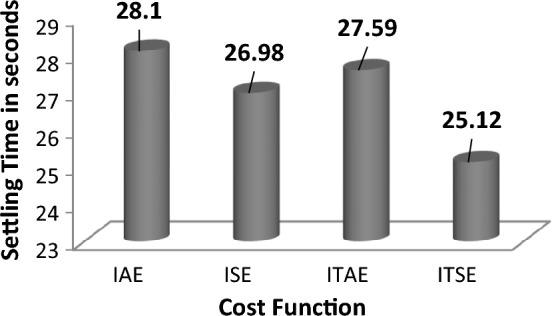
Settling time comparisons for ASIA-tuned FOPID controller with different cost functions.

The response comparisons of ASIA technique-tuned FOPID controller response with different cost function in area 1 is shown in Fig. 18. The numerical values of settling with different cost bar chart comparisons are shown in Fig. 19.

By examining the response comparisons in Fig. 18 and bar chart comparisons in Fig. 19, ITSE cost function-based ASIA FOPID controller provides better controller action over IAE, IAE and ITAE functions-based ASIA FOPID controller. Also, the system performance is improved using ITSE cost function-based ASIA FOPID controller by achieving minimal settling time during sudden load demand. Moreover, Table 12 validate the Percentage of improvement of ITSE-based SCIA-PID controller over other cost functions.

**Table 12:** Percentage of improvement of ITSE-based ASIA-FOPID controller over other cost functions.

Cost function	Relaxation time (s)	Percentage of improvement (%)
IAE	28.1	10.60
ISE	26.98	6.89
ITAE	27.59	8.95

### Analysis of the proposed controllers' robustness by changing system parameters and load pattern

In this section, the robustness of the suggested optimization techniques and controllers are examined by changing system parameters ± 25% and ± 50% from its nominal value and the load pattern change by 1%, 2%, 5% and 10%. The details are given in the following two sub-sections.

1. System parameter change

In this section governor and turbine time constants are changed from their nominal value. The behavior comparisons are given in Figs. 20, 21, 22, 23, 24, 25, 26, and 27.

**Figure 20:**
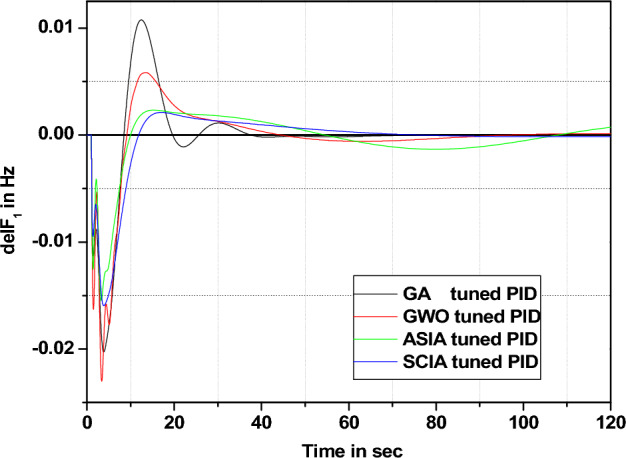
Area 1 Frequency deviation comparisons of GA, GWO, ASIA, SCIA tuned PID controller response with turbine time constant changes (− 25%) from its nominal value.

**Figure 21:**
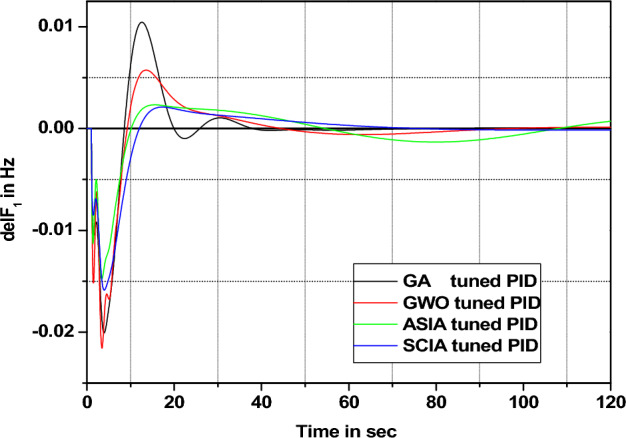
Area 1 Frequency deviation comparisons of GA, GWO, ASIA, SCIA tuned PID controller response with turbine time constant changes (− 50%) from its nominal value.

**Figure 22:**
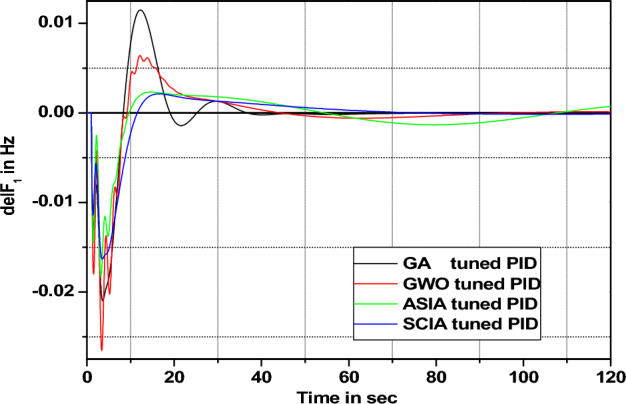
Area 1 Frequency deviation comparisons of GA, GWO, ASIA, SCIA tuned PID controller response with turbine time constant changes (+ 25%) from its nominal value.

**Figure 23:**
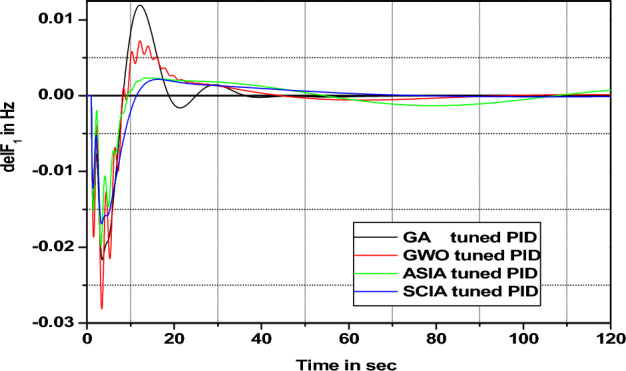
Area 1 Frequency deviation comparisons of GA, GWO, ASIA, SCIA tuned PID controller response with turbine time constant changes (+ 50%) from its nominal value.

**Figure 24:**
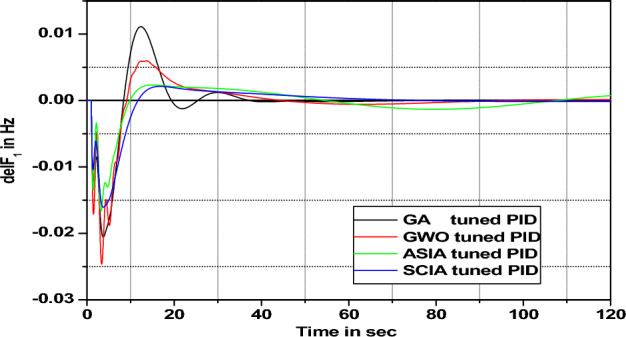
Area 1 Frequency deviation comparisons of GA, GWO, ASIA, SCIA tuned PID controller response with governor time constant changes (− 25%) from its nominal value.

**Figure 25:**
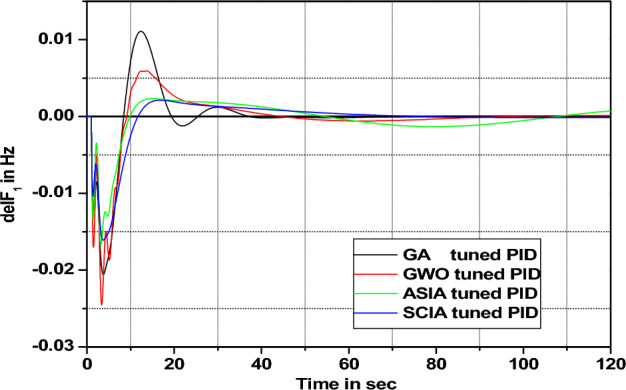
Area 1 Frequency deviation comparisons of GA, GWO, ASIA, SCIA tuned PID controller response with governor time constant changes (− 50%) from its nominal value.

**Figure 26:**
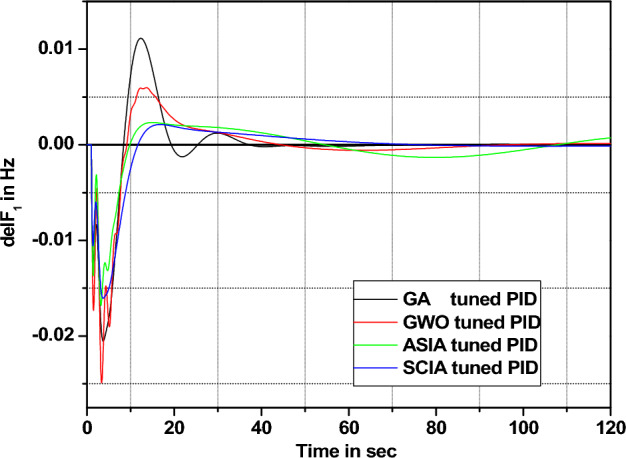
Area 1 Frequency deviation comparisons of GA, GWO, ASIA, SCIA tuned PID controller response with governor time constant changes (+ 25%) from its nominal value.

**Figure 27:**
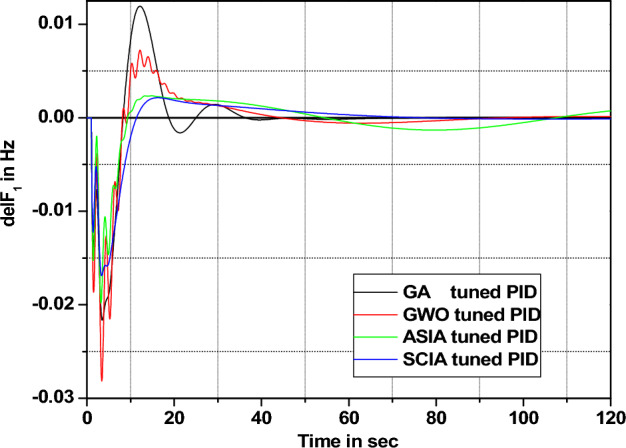
Area 1 Frequency deviation comparisons of GA, GWO, ASIA, SCIA tuned PID controller response with governor time constant changes (+ 50%) from its nominal value.

Figures 20, 21, 22, and 23 show response comparisons of GA, GWO, ASIA and SCIA techniques tuned controller performance when turbine time constant changes from its nominal value. It is effectively indicating that the proposed optimization techniques are efficient under system parameter change scenarios.

Figures 24, 25, 26, and 27 show the response (are 1 frequency deviations) comparisons of GA, GWO, ASIA and SCIA techniques tuned PID controller performance when governor time constant values change in the analyzed power system.

The performance comparisons of ASIA and SCIA techniques tuned FOPID controller subjected to turbine and governor time constant values change ± 25% and ± 50% from its nominal values are given in Fig. 28, 29, 30, 31, 32, 33, 34, and 35.

**Figure 28:**
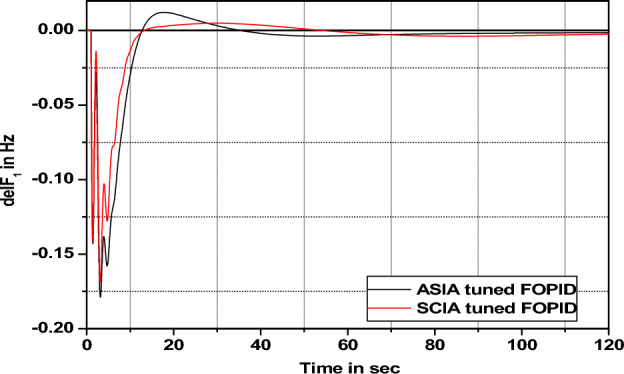
Area 1 Frequency deviation comparisons of ASIA, SCIA tuned FOPID controller response with governor time constant changes (− 25%) from its nominal value.

**Figure 29:**
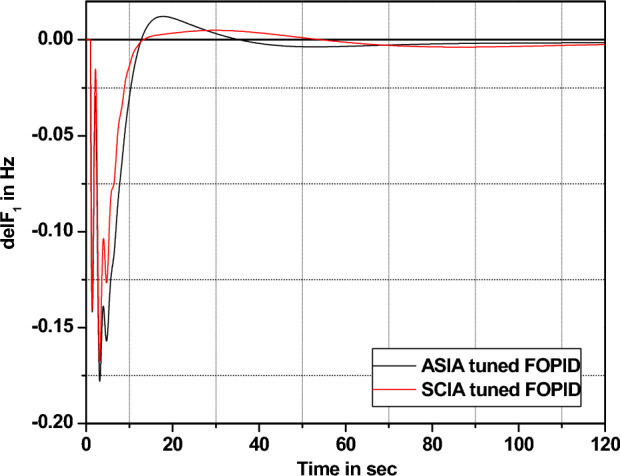
Area 1 Frequency deviation comparisons of ASIA, SCIA tuned FOPID controller response with governor time constant changes (− 50%) from its nominal value.

**Figure 30:**
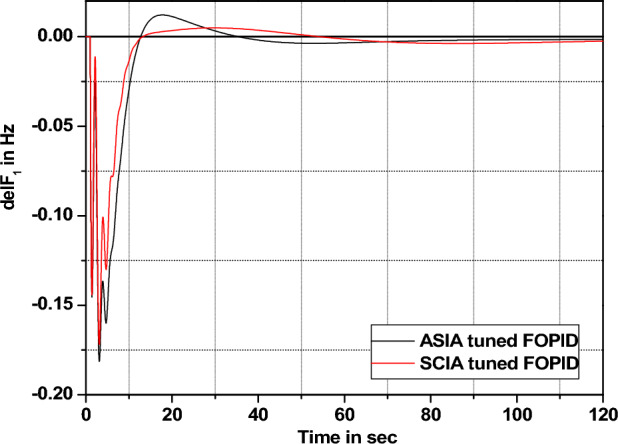
Area 1 Frequency deviation comparisons ASIA, SCIA tuned FOPID controller response with governor time constant changes (+ 25%) from its nominal value.

**Figure 31:**
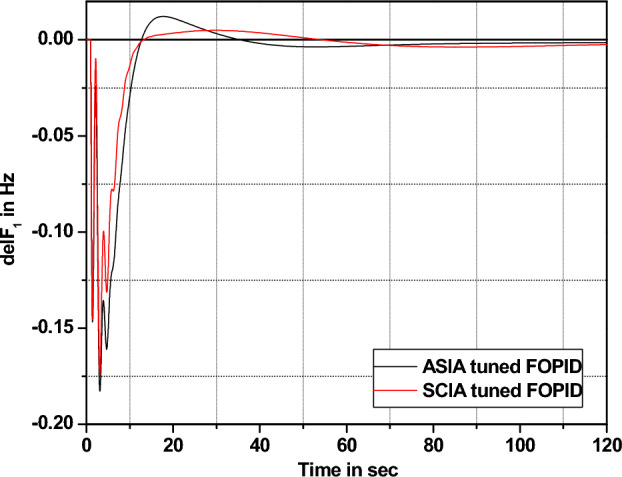
Area 1 Frequency deviation comparisons of ASIA, SCIA tuned FOPID controller response with governor time constant changes (+ 50%) from its nominal value.

**Figure 32:**
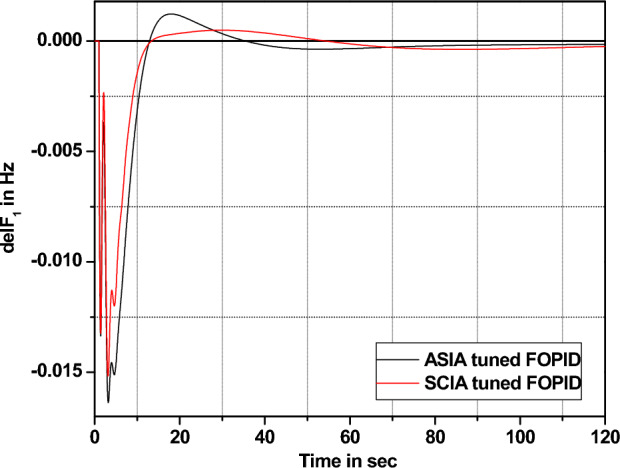
Area 1 Frequency deviation comparisons of ASIA, SCIA tuned FOPID controller response with turbine time constant changes (− 25%) from its nominal value.

**Figure 33:**
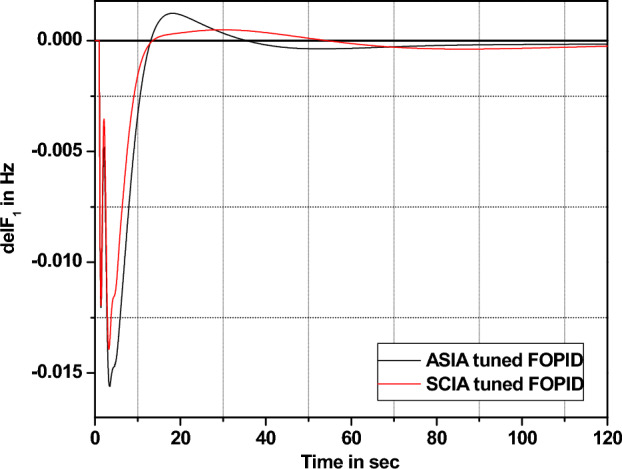
Area 1 Frequency deviation comparisons of ASIA, SCIA tuned FOPID controller response with turbine time constant changes (− 50%) from its nominal value.

**Figure 34:**
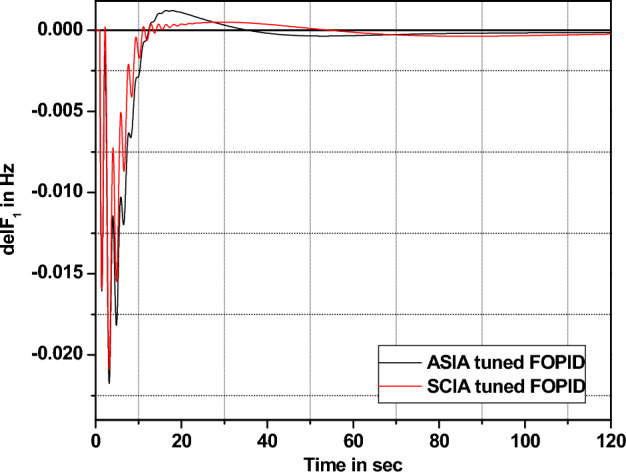
Area 1 Frequency deviation comparisons ASIA, SCIA tuned FOPID controller response with turbine time constant changes (+ 25%) from its nominal value.

**Figure 35:**
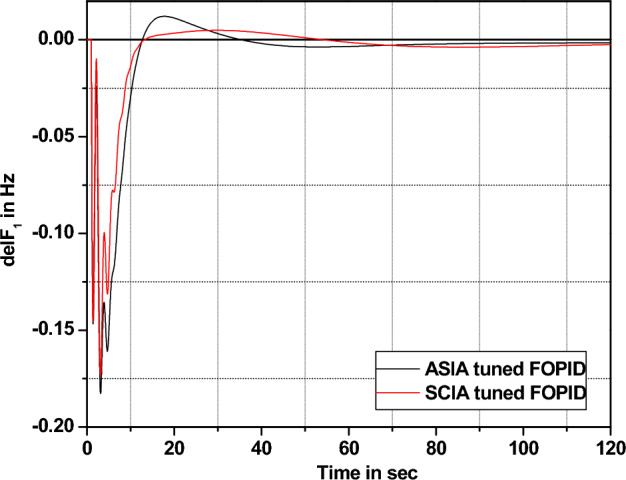
Area 1 Frequency deviation comparisons of ASIA, SCIA tuned FOPID controller response with turbine time constant changes (+ 50%) from its nominal value.

Based on the above robustness test analysis (Figs. 20, 21, 22, 23, 24, 25, 26, 27, 28, 29, 30, 31, 32, 33, 34, and 35), it is found that the analyzed optimization techniques (GA, GWO, ASIA and SCIA) and controller (PID and FOPID) provide better control action when system parameter changes occur from its nominal value (± 25%, ± 50%). In this analysis turbine and governor time constants variations are considered for investigations.

2. Changing load pattern

**Figure 36:**
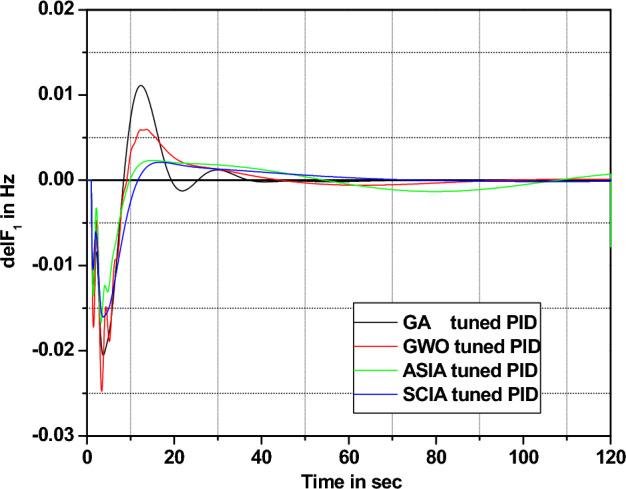
Area 1 Frequency deviation comparisons of GA, GWO, ASIA, SCIA tuned PID controller response with 1% SLP.

**Figure 37:**
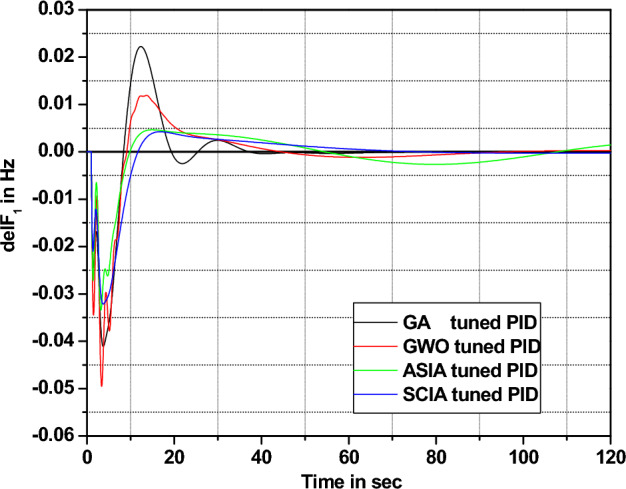
Area 1 Frequency deviation comparisons of GA, GWO, ASIA, SCIA tuned PID controller response with 2% SLP.

**Figure 38:**
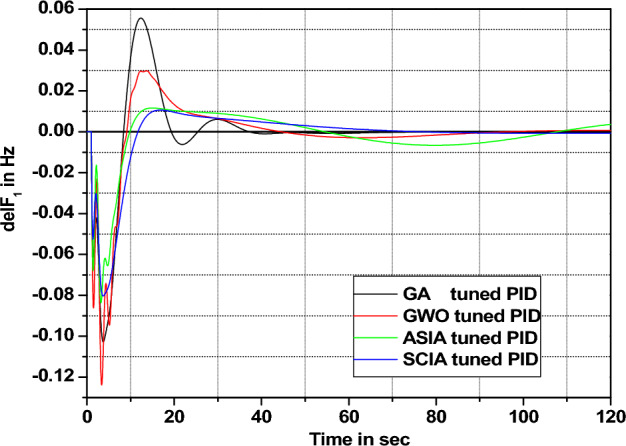
Area 1 Frequency deviation comparisons of GA, GWO, ASIA, SCIA tuned PID controller response with 5% SLP.

**Figure 39:**
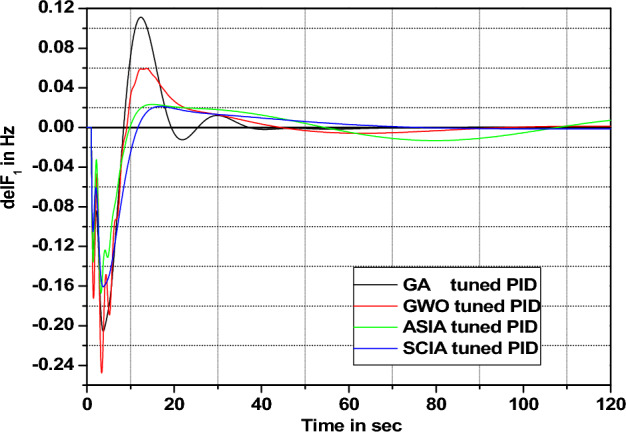
Area 1 Frequency deviation comparisons of GA, GWO, ASIA, SCIA tuned PID controller response with 10% SLP.

**Figure 40:**
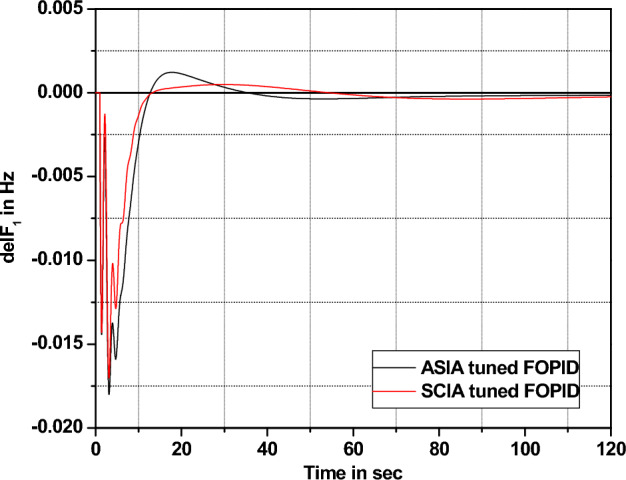
Area 1 Frequency deviation comparisons of ASIA, SCIA tuned FOPID controller response with 1% SLP.

**Figure 41:**
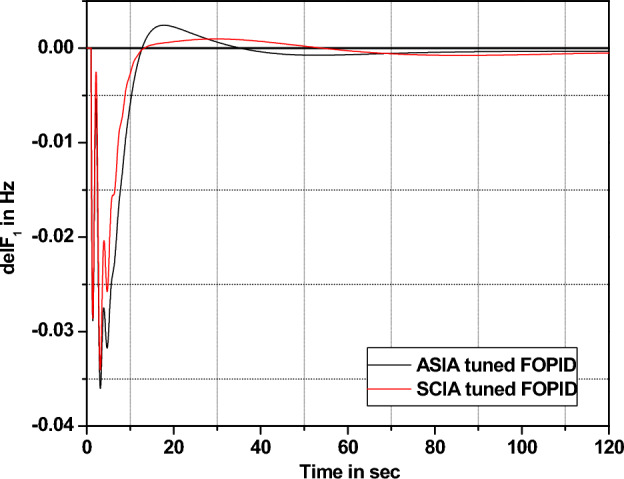
Area 1 Frequency deviation comparisons of ASIA, SCIA tuned FOPID controller response with 2% SLP.

**Figure 42:**
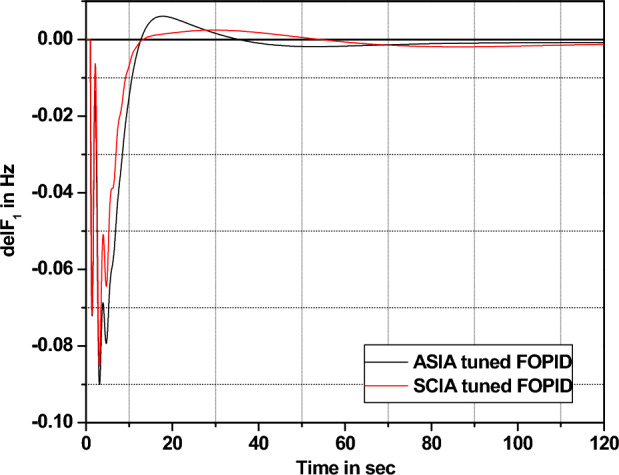
Area 1 Frequency deviation comparisons of ASIA, SCIA tuned FOPID controller response with 5% SLP.

**Figure 43:**
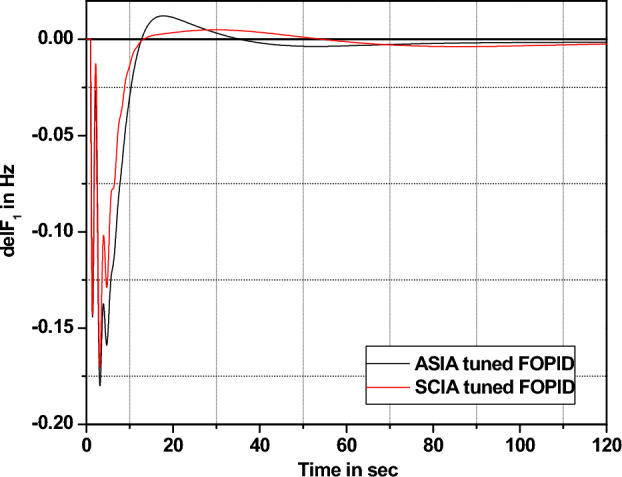
Area 1 Frequency deviation comparisons of ASIA, SCIA tuned FOPID controller response with 10% SLP.

The effectiveness of both proposed optimization techniques and controllers is examined by applying different load patterns for both PID and FOPID controllers. The performance comparisons are depicted in Figures 36, 37, 38, 39, 40, 41, 42, and 43. The response of the system equipped with PID controller tuned by the suggested optimization techniques subjected to different load patterns is elaborated in figures 36, 37, 38, and 39.

Figures 40, 41, 42, and 43 indicate the response comparisons of ASIA and SCIA techniques-based FOPID controllers under different loading patterns (1%, 2%, 5% and 10%).

The main observation of the above robustness analysis (Figs. 36, 37, 38, 39, 40, 41, 42, and 43) originates that the analyzed ASIA and SCIA optimization techniques based on FOPID controllers provide better control action under different load patterns situations. In this analysis, the suggested well tunned FOPID controllers provide and yield better controller action in the investigated system.

## Conclusion

In this work, PID and FOPID controllers have been projected as secondary controllers for three area thermal-hydro-wind interconnected power generating systems. The optimized gain values of PID controller are obtained by implementing GA, GWO, SCIA and ASIA methods with four types of cost functions namely, IAE, ITAE, ISE and ITSE.

Based on the simulation results, it is observed that ITSE cost function gives a fast settled response in GWO-PID controller response and it improves the performance of the system by 46.01%, 37.37% and 71.64% over IAE, ISE and ITAE cost functions based GWO-PID controllers, respectively.

Similarly, ISE-based GA-PID controller performance yields superior performance compared to other cost functions tuned controllers by 25.93%, 12.49% and 14.11% over IAE, ITAE and ITSE-based GA-PID controller performance, respectively.

The simulation results depict that ITSE cost function based ASIA optimized controller (PID) yields better performance over the remaining cost functions employing better time domain parameters and also it improves the response of the system by 53.44%, 52.71% and 29.18% over IAE, ISE and ITAE cost functions-based controllers, respectively. Moreover, ITSE-based ASIA-FOPID controller provides superior response over other cost functions (10.60%, 6.89% and 8.95% over IAE, ISE and ITAE).

Likewise, the different cost functions-based SCIA technique tuned PID controller behavior demonstrates that ISE cost function-based SCIA-PID controller is superior to other cost functions based controllers (23.03%, 17.61% and 27.48% over IAE, ITAE and ITSE). Correspondingly, ISE cost function-based FOPID controller tuned by SCIA provides better control action over the remaining cost functions-based controller response (3.54%, 2.06% and 1.08% over IAE, ITSE and ITAE).

This proposed article indicates that the proposed ISE cost function-based GA- PID, SCIA – PID and FOPID controllers in addition to ITSE-based GWO-PID, ASIA-PID and FOPID controllers outperformed well than other cost functions based controllers for multi-area interlinked power system with renewable power plant even in the case of system parameters change and different loading conditions.

## Data Availability

The authors would like to confirm that all data generated or analyzed during this study are included in this published article.
